# Translating mouse models of abdominal aortic aneurysm to the translational needs of vascular surgery

**DOI:** 10.1016/j.jvssci.2021.01.002

**Published:** 2021-03-03

**Authors:** Albert Busch, Sonja Bleichert, Nahla Ibrahim, Markus Wortmann, Hans-Henning Eckstein, Christine Brostjan, Markus U. Wagenhäuser, Craig J. Goergen, Lars Maegdefessel

**Affiliations:** a Department for Vascular and Endovascular Surgery, Technical University Munich, Munich, Germany; b Deutsches Zentrum für Herz-Kreislaufforschung (DZHK), Berlin, Germany; c Division of Vascular Surgery and Surgical Research Laboratories, Department of Surgery, Medical University of Vienna, Vienna General Hospital, Vienna, Austria; d Department of Vascular and Endovascular Surgery, Universitaetsklinik Heidelberg, Heidelberg, Germany; e Department of Vascular and Endovascular Surgery, Heinrich-Heine-University Medical Center Düsseldorf, Düsseldorf, Germany; f Weldon School of Biomedical Engineering, Purdue University, West Lafayette, Ind

**Keywords:** Abdominal aortic aneurysm, AAA, Aneurysm mouse models, Translational research

## Abstract

**Introduction:**

Abdominal aortic aneurysm (AAA) is a condition that has considerable socioeconomic impact and an eventual rupture is associated with high mortality and morbidity. Despite decades of research, surgical repair remains the treatment of choice and no medical therapy is currently available. Animal models and, in particular, murine models, of AAA are a vital tool for experimental in vivo research. However, each of the different models has individual limitations and provide only partial mimicry of human disease. This narrative review addresses the translational potential of the available mouse models, highlighting unanswered questions from a clinical perspective. It is based on a thorough presentation of the available literature and more than a decade of personal experience, with most of the available models in experimental and translational AAA research.

**Results:**

From all the models published, only the four inducible models, namely the angiotensin II model (AngII), the porcine pancreatic elastase perfusion model (PPE), the external periadventitial elastase application (ePPE), and the CaCl_2_ model have been widely used by different independent research groups. Although the angiotensin II model provides features of dissection and aneurysm formation, the PPE model shows reliable features of human AAA, especially beyond day 7 after induction, but remains technically challenging. The translational value of ePPE as a model and the combination with β-aminopropionitrile to induce rupture and intraluminal thrombus formation is promising, but warrants further mechanistic insights. Finally, the external CaCl_2_ application is known to produce inflammatory vascular wall thickening. Unmet translational research questions include the origin of AAA development, monitoring aneurysm growth, gender issues, and novel surgical therapies as well as novel nonsurgical therapies.

**Conclusion:**

New imaging techniques, experimental therapeutic alternatives, and endovascular treatment options provide a plethora of research topics to strengthen the individual features of currently available mouse models, creating the possibility of shedding new light on translational research questions.

Abdominal aortic aneurysm (AAA) is a disease of great socioeconomic importance and has substantial repercussions on the individual patient, especially in the case of rupture with an imminent threat to a patient's life.^1^ It is by far the most frequent type of aortic aneurysm.^2^ Currently, the treatment is exclusively surgical and based on a certain diameter threshold or growth rate. No medical treatment for prevention or stabilization of AAAs exists, despite a variety of clinical trials in the past years (reviewed in detail by Lindeman et al^3^).^4^^,^^5^ Thus, the need for better translational research addressing these aims is crucial.

In recent years, organoids and organs-on-a-chip technology that mimics complex human tissues and diseases have become an interesting alternative for in vivo experiments.^6^ However, no such alternative models are available for aortic research (yet). Thus, animal models remain the gold standard for translational research. In this review, we elaborate on the mechanisms of commonly used murine AAA models and their potential application in a translational setting to address questions of modern daily medical practice dealing with patients with AAA.

## History of AAA mouse models

In the last three decades, a variety of animal models have been presented for AAA research and each of them has been successfully transferred to the mouse (Table I). Mice are one of the most frequently used laboratory animals owing to inexpensive housing, established research methods, genetic resemblance/engineering, and accessibility.^17^ In chronological order, we will introduce the angiotensin II infusion model in hyperlipidemic (most commonly *ApoE*^*-/-*^) mice (AngII), the porcine pancreatic elastase perfusion model (PPE), the calcium chloride model (CaCl_2_), and the external periadventitial elastase application model (ePPE). We then examine the effect β-aminopropionitrile enhancement in two of these models (+BAPN) (Table I).^7^, ^8^, ^9^, ^10^, ^11^, ^12^

**Table I:** Overview of abdominal aortic aneurysm (AAA) mouse models

	First description in mouse	No. of publications (approximately)	No. of independent groups publishing (approximately)
Inducible			
AngII model (+BAPN)	Daugherty et al, 2000^7^ Kanematsu et al, 2010^8^	>10014	>107
PPE model	Pyo et al, 2000^9^	26	10
CaCl_2_ model	Chiou et al, 2001^10^	67	>10
ePPE model (+BAPN)	Bhamidipati et al, 2012^11^ Lu et al, 2017^12^	42	62
Spontaneous			
Blotchy/mottled mice	Andrews et al, 1975^13^	7	3-4
ApoE^−/−^ or LdlR^−/−^ mice on a HFD	Tangirala et al, 1995^14^	16	13
+Timp1 double KO	Silence et al, 2002^15^	3	2
+eNOS double KO	Kuhlencordt et al, 2001^16^	2	1

*AngII*, Angiotensin II; *ApoE*, apolipoprotein E; *BAPN*, ß-aminopropionitrile; *eNOS*, endothelial nitric oxide synthase; *HFD*, high-fat diet; *KO*, knockout; *Ldlr*, low-density lipoprotein receptor; *PPE*, periadventitial elastase application; *Timp1*, tissue inhibitor of metalloproteinase 1.

The number of publications and the number of independent groups publishing is based on a term-specific search on PubMed. Independent groups are defined as research groups differing in principal investigator and location or are personally known to the authors. AngII and ePPE have been demonstrated with additional abdominal aortic aneurysm-specific features when coadministered with BAPN (see Table II).

Generally speaking, spontaneous models cannot be considered readily applicable owing to low penetrance of the phenotype and/or a heterogeneous distribution of the lesion location.^18^ Hence, this review focuses on the commonly used inducible AAA models (Table I).

Further models with some scientific impact have been introduced, but have either not been transferred to mice or not reached broad applicability. One example is the decellularized aortic xenograft model, where the infrarenal aorta is exchanged by orthotopic transplantation in between species or genetically modified animals.^19^^,^^20^ This technique has been shown to be feasible in mice as well; however, no further scientific reports have followed.^21^ Another example is the administration of deoxycorticosterone acetate plus BAPN or additional salt.^8^^,^^22^ Over the years, modifications of these inducible models have been performed and reported in rats, hamsters, guinea pigs, rabbits, turkeys, sheep, dogs, and pigs.^23^

## Pathomechanisms of frequently used models in relation to human disease

Although the mouse and human genome do possess large similarities, there are physiologic processes that mice cannot replicate and might be better mimicked in rats.^17^^,^^24^ Good evidence for the shortcomings of mouse models comes from cancer research with a minimal translation rate of an average of 1 in 1000 drugs proceeding from preclinical testing to clinical trials.^25^

**Fig 1:**
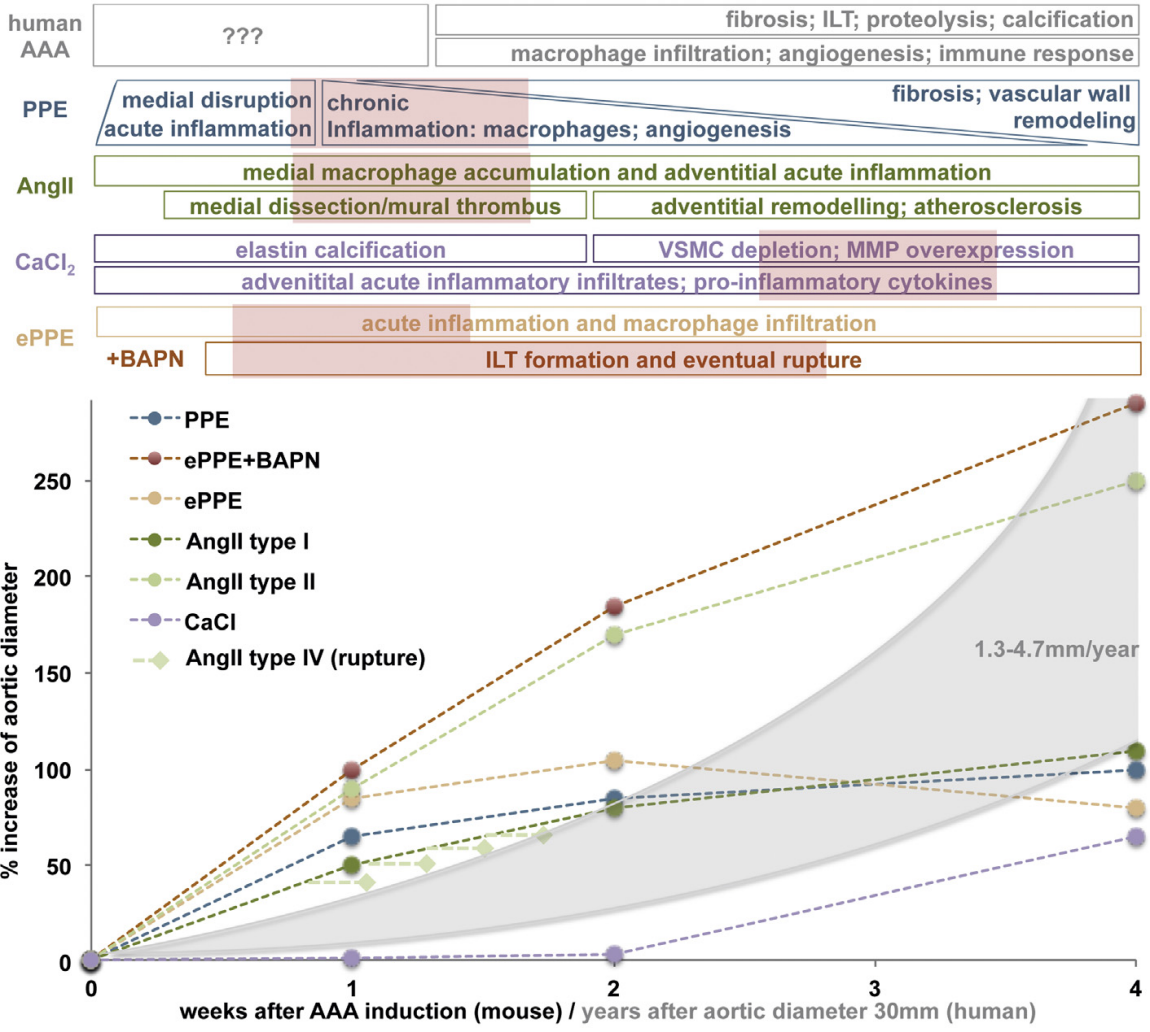
Timeline of abdominal aortic aneurysm (*AAA*) development in mouse models. Although the data for aortic diameter enlargement are reported for different time points in many studies, the specific molecular characteristics of the aortic wall are mainly reported for the time of sacrifice and based on the specific study. In human disease, the timeline of events is largely unclear, since samples are only available from the time of surgery, normally when diameter exceeds 50 mm and also the initial stimulus for abdominal aortic aneurysm (*AAA*) development is completely unknown. However, human AAA tends to grow exponentially based on diameter (grey area) in a chronic manner.^4^ This graph shows the percentage growth of the aortic diameter for the first 4 weeks after AAA induction for various mouse models. This data are based on the available systematic reviews and leading articles and are semiquantitative only to compare aortic enlargement.^12^^,^^29^, ^30^, ^31^, ^32^ For external periadventitial elastase application (*ePPE*), the addition of β-aminopropionitrile (BAPN) (red line) results in a marked increase in aneurysm diameter. For the angiotensin II model (*AngII*), the classification suggested by Daugherty et al^29^ in 2011 (see text) is included: type I (dark green; dilation <2 times baseline) and type II (light green: dilation <2 times baseline); type IV (light green rhomb: rupture) can occur at any time, most likely within days 4 to 10 after minipump implantation. The timeline of events in the aortic wall in comparison with the features of human disease can only be assumed for many of the models and specific details warrant further elucidation. The red boxes suggest time frames for interventional studies on AAA mouse models to suggest that not only initial stimulus-based, but human disease mimicking mechanisms are being interfered with. For most models, some aortic diameter data beyond 4 weeks after aneurysm induction is available (for ≤10 weeks) and demonstrates further flattening of the growth curve (not included in this figure). *ILT*, Intraluminal thrombus; *VSMC*, vascular smooth muscle cell.

Considering these shortcomings, it is essential to thoroughly evaluate models based on a profound knowledge of their specific limitations to produce high-quality data and their potential for continuous improvement. To this end, the proposed mechanism of action, the timeline of AAA development and the mimicry of human disease of the most established, inducible AAA mouse models in the field are presented in Table II and Fig 1. The specific pathomechanisms of AAA mouse models have been extensively reviewed in detail many times before and will only briefly be summarized for their main characteristics.^10^^,^^11^^,^^29^^,^^33^^,^^34^

**Table II:** Features of human abdominal aortic aneurysm (*AAA*) reflected in mouse models

Feature of human AAA	Ang II	PPE	CaCl_2_	EPPE
Fibrosis	x	x	x	X
Fusiform aneurysm growth	- Frequency increased by BAPN	x	x	X Diameter increased by BAPN
Aortic dissection	x Frequency increased by BAPN	-	-	-
ILT	-	(x)	-	X
Intramural hemorrhage	x	-	-	-
Altered hemodynamics	x	x	-	X
Imbalanced proteolysis	x	x	x	X
Angiogenesis	x	x	x	-
Humoral immune response	X	x	-	-
Calcification	-	-	x	-
Rupture	x (early)	-	-	x (late) only induced by BAPN

*x*, Has been described; –, not yet described; *AngII*, angiotensin II; *BAPN*, ß-aminopropionitrile; *ILT*, intraluminal thrombus; *PPE*, periadventitial elastase application.

The specific features of human AAA considered important have been reviewed by others and us before.^26^, ^27^, ^28^ The table shows if these have been reported in the inducible mouse models. Additionally, the effect of BAPN on the specific aneurysm feature is described.

### Pathomechanism of AngII

Although based on the initial description of aortic lesions in high salt intake-induced angiotensin II overproducing mice by Nishijo et al^35^ in 1998, the model as we know it today is attributed to Daugherty et al,^7^ who implanted osmotic minipumps and published on “Unexpectedly, pronounced abdominal aortic aneurysms were present in *ApoE*^*−/−*^ mice infused with Ang II” in 2000 while looking for a way to increase blood pressure in atherosclerotic mouse models.^7^^,^^35^

Mechanistically, early medial disruption at the suprarenal aorta with subsequent dissection, nonfusiform lumen enlargement, neutrophil recruitment and secondary hemorrhage occur over a relatively predictable timeline. After 10 to 14 days, continuous remodeling with characteristic features of pronounced leukocyte infiltration of macrophages, T and B lymphocytes, collagen deposition, and neovascularization occurs as a more chronic phase.^29^ Complex flow patterns form within the dissection and likely have an influence on thrombus deposition and inflammatory cell infiltrate.^36^ Why this affects the suprarenal portion of the abdominal aorta in mice remains unknown and needs to be considered in AAA research as the infrarenal and suprarenal regions of the aorta might have different embryological backgrounds.^29^^,^^37^

**Fig 2:**
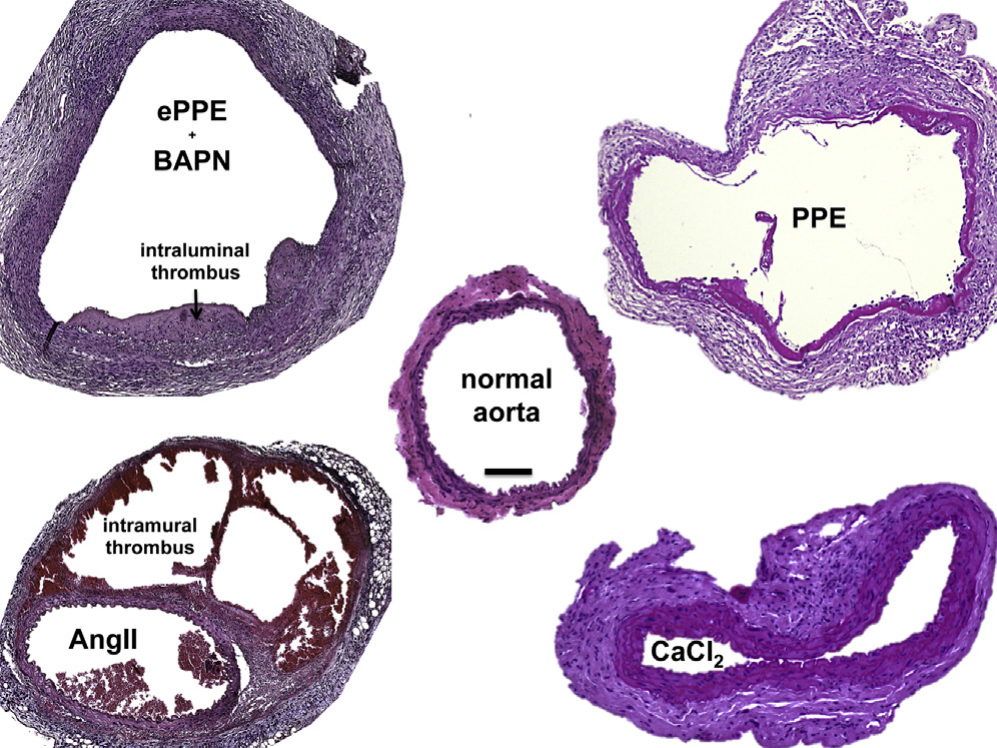
Histologic review of inducible abdominal aortic aneurysm (*AAA*) murine models. Histologic cross-sections from 28 days after aneurysm induction are presented with hematoxylin and eosin staining. The normal mouse infrarenal aorta is approximately 500 μm in diameter when perfused and contains a medial layer with four to five elastic lamellar units. One layer of endothelial cells lines the inner luminal layer, and a surrounding adventitia is composed of mainly connective tissue. In the external periadventitial elastase application (*ePPE*) (β-aminopropionitrile [+BAPN]) model, the adventitia and media show cellular enrichment and the medial elastin breakdown. Most notably is the intraluminal thrombus (ILT). In contrast, for the angiotensin II model (*AngII*) aorta, the media remains mostly intact and cellular enrichment is more prominent in the adventitia. Note the thrombus formation in between the media and the adventitia (washed out in parts owing to fixation). In the PPE model, the media is mostly disrupted, and the adventitia shows increased fibrosis, signs of chronic inflammation and angiogenesis (not shown). Finally, in the CaCl_2_ model, the elastic fibers remain intact but thicken along with the adventitia and exhibits signs of fibrosis and inflammatory infiltrates in all layers. Scale bar = 100 μm; original magnification ×10; histologic images are courtesy of the authors.
• Type II denotes a large single dilation (> 2 times of a normal diameter) (Fig 1)
• Type III categorizes multiple dilations
• Type IV classifies aortic rupture that leads to death owing to bleeding into the peritoneal or thoracic cavities (Fig 1)

In 2015, Trachet et al^30^ performed a meta-analysis of 143 treatment studies involving the AngII model from 2000 to 2015. In a multivariate analysis, they identified that sex, age, genetic background, infusion time, and dose of angiotensin II significantly influence AAA incidence. They also showed that animal sex, genetic background and dose influenced rupture-associated mortality. Hence, in 2011, Daugherty et al^29^ suggested a reporting standard for their model defining the type of lesion:

• Type I represents a small single dilation (1.5-2.0 times of a normal diameter) (Figs 1 and 2)

And reporting on three different experimental readouts^29^:

• Rupture rate of the dissected aorta
• Overall survival
• AAA/dissection diameter

### Pathomechanism of PPE

**Fig 3:**
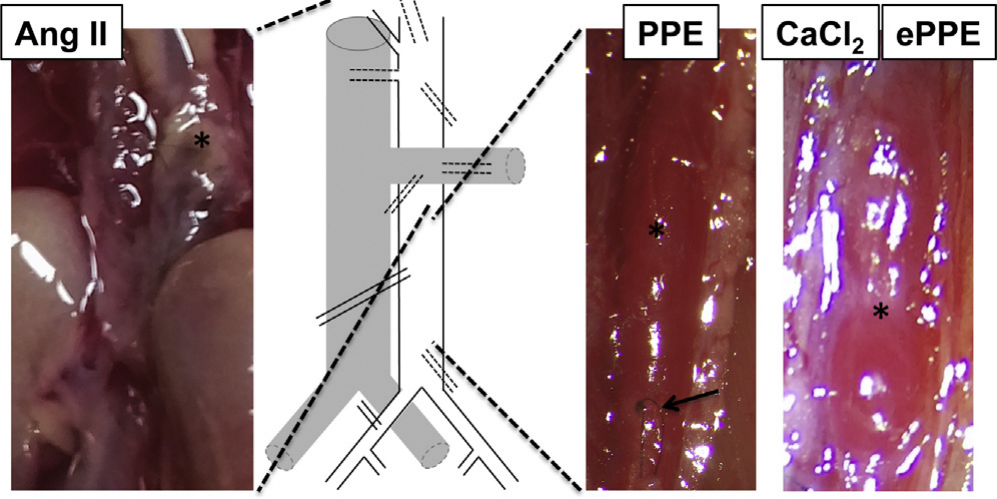
Macroscopic review of inducible abdominal aortic aneurysm (*AAA*) murine models. The photographs reveal the aneurysm (∗) of individual models in situ. In the angiotensin II model (*AngII*), the maximum dilation occurs at the thoracoabdominal and visceral sections of the aorta (dashed lines). In the other models, the exact formation of aneurysm is due to the site of exposure of the aorta. Note the suture (*arrow*) from elastase perfusion in PPE and the vast retroperitoneal adhesion with the surrounding tissue in the CaCl_2_ and the external periadventitial elastase application (*ePPE*) (photo shown) model.

This surgically challenging model was first performed and described by Anidjar et al^38^ in 1990 in rats and later translated to mice by Pyo et al in 2000.^9^ An isolated part of the aorta is intraluminally pressure perfused with porcine pancreatic elastase via a catheter (discussed elsewhere in this article). This results in a degradation of the medial elastic lamellae, with subsequent inflammatory response, followed by aneurysmal dilatation (Figs 2 and 3). However, the exogenously infused elastase is undetectable 48 hours after infusion, and heat-inactivated elastase does not cause aneurysms.^39^ Increased expression of various matrix metalloproteinases (MMPs), cathepsins and other proteases was observed, and chemical or genetic depletion of MMP activity was shown to halt aneurysm induction, suggesting a macrophage-dependent increased elastolysis and extracellular matrix turnover.^9^^,^^40^ Interestingly, the plasminogen-urokinase-plasmin axis seems to be critically involved in aneurysm formation, placing a certain emphasis on elastin breakdown products, such as collagen XIII and endostatin.^41^^,^^42^

(Individual photographs are courtesy of the authors.)

Upstream of increased MMP production, the leukotriene pathway is believed to enhance aneurysm formation and antiasthmatic therapies have been successfully tested in the PPE model.^43^^,^^44^ The model can be performed on basically any mouse strain and the phenotype can be enhanced by additional external stimuli, such as nicotine.^45^

The main limitations are the microsurgical challenge of manipulating the 500-μm murine aorta and the fact that no intraluminal thrombus (ILT) is observed in this model. Additionally, there is a considerable rate of hind limb ischemia owing to the aortic cross-clamping, resulting in either intraluminal thrombosis or spinal cord ischemia. Unfortunately, a systematic review of this model is currently missing.

### Pathomechanism of CaCl_2_

Initially designed to induce Prinzmetal angina-like vasospasm, Gertz et al^46^ used CaCl_2_ topically in the rabbit carotid artery not finding the anticipated stenosis, but a diameter enlargement after 3 days. Mechanistically, a CaCl_2_-elastin complex subsequently attracting inflammatory leukocytes is described after applying 0.25 to 0.5 mol/L CaCl_2_ topically for 15 minutes. The model was slightly modified by Yamanouchi et al,^47^ by applying additional PBS after the CaCl2 procedure, hence adding phosphate to produce larger aortic dilation along calcifications. This model develops fusiform infrarenal AAAs, but with more vascular wall thickening (Figs 2 and 3). This can sometimes lead to discrepancies between ex vivo measurements where outer diameter is measured and in vivo imaging approaches where lumen diameter is typically quantified (See the Critical appraisal section).

In 2012, Wang et al^31^ reviewed 29 studies applying the model, thereof 19 in mice, and summarized the variations in technical approaches and outcomes with respect to size and penetrance of AAA lesions.

### Pathomechanism of ePPE

The topical application of elastase on the murine aorta was introduced by Bhamidipati et al in 2012.^11^ They applied porcine pancreatic elastase at a higher concentration than the typical PPE model to the ventral aorta, and hence did not have to dissect or clamp the aorta. The changes observed include acute inflammation and macrophage infiltration, followed by aortic dilation. Despite there often being a misunderstanding of the common pathophysiology of intraluminal and topically administered PPE, our group was able to demonstrate distinct forms of inflammation in both models and a much higher concentration of elastase needed when applied from the outside (Fig 2).^33^ In the latter, we see severe ongoing acute inflammatory infiltrates resulting in excessive retroperitoneal inflammation in the animals. With the ePPE model being the newest, no conclusive review or meta-analysis is currently available.

### Addition of BAPN

The lysyl oxidase inhibitor BAPN blocks crosslinking of elastin and collagen and thereby reduces matrix stability. It was inadvertently found to increase the rate of AAA in mice that were coadministered AngII in a study of hypertensive disease in 2010.^8^ Similarly, the penetrance of thoracic aortic dissection increased to 100% with the addition of BAPN in the AngII model.^48^ In combination with ePPE, BAPN in the drinking water increases the AAA diameter and can also induce rupture.^12^^,^^34^ In this model, no dissections are observed, in contrast with the results seen in combination with the AngII model (Figs 1 and 2).^49^ BAPN alone was shown to induce thoracic aortic dissection in juvenile mice in a dose-dependent manner and is frequently used as a model for thoracic aortic dissection.^50^

Additionally, combinations of the different inducible AAA model techniques have been described, but have not been evaluated clearly by the research community, with only preliminary studies describing AngII in combination with CaCl_2_.^51^

### The significance of the atherosclerotic murine aorta

Arterial occlusive disease and AAA share some of the same risk factors, but are considered distinct pathologies in humans.^26^^,^^52^ In mice, atherosclerosis in the susceptible portions of the aorta likely plays some role in most of the presented models. This is achieved by either specific knockouts leading to hypercholesterolemia (ie, *ApoE*^-/-^ or *Ldlr*^-/-^) or a diet rich in saturated fatty acid (Western or high-fat diet).

Although hypercholesterolemia is not conditio sine qua non, it increases the incidence of the AAA/dissection phenotype in AngII by three to four times.^29^ The AngII model can also be used in C57BL/6 wild-type mice, but the frequency of the dissecting lesions is much lower.^53^ Similarly, the high-fat diet increases the rupture rate in AngII mice.^30^ Additional high salt intake (NaCl) does not increase the lesion diameter, but worsens the signs of extracellular matrix remodeling within the aortic wall and may affect the rupture rate.^22^^,^^54^

Although best investigated for the AngII model, in general, the phenotype of the model-specific lesions can be highlighted by adding additional stress to the murine aorta.

## Technical considerations for inducible AAA models

**Fig 4:**
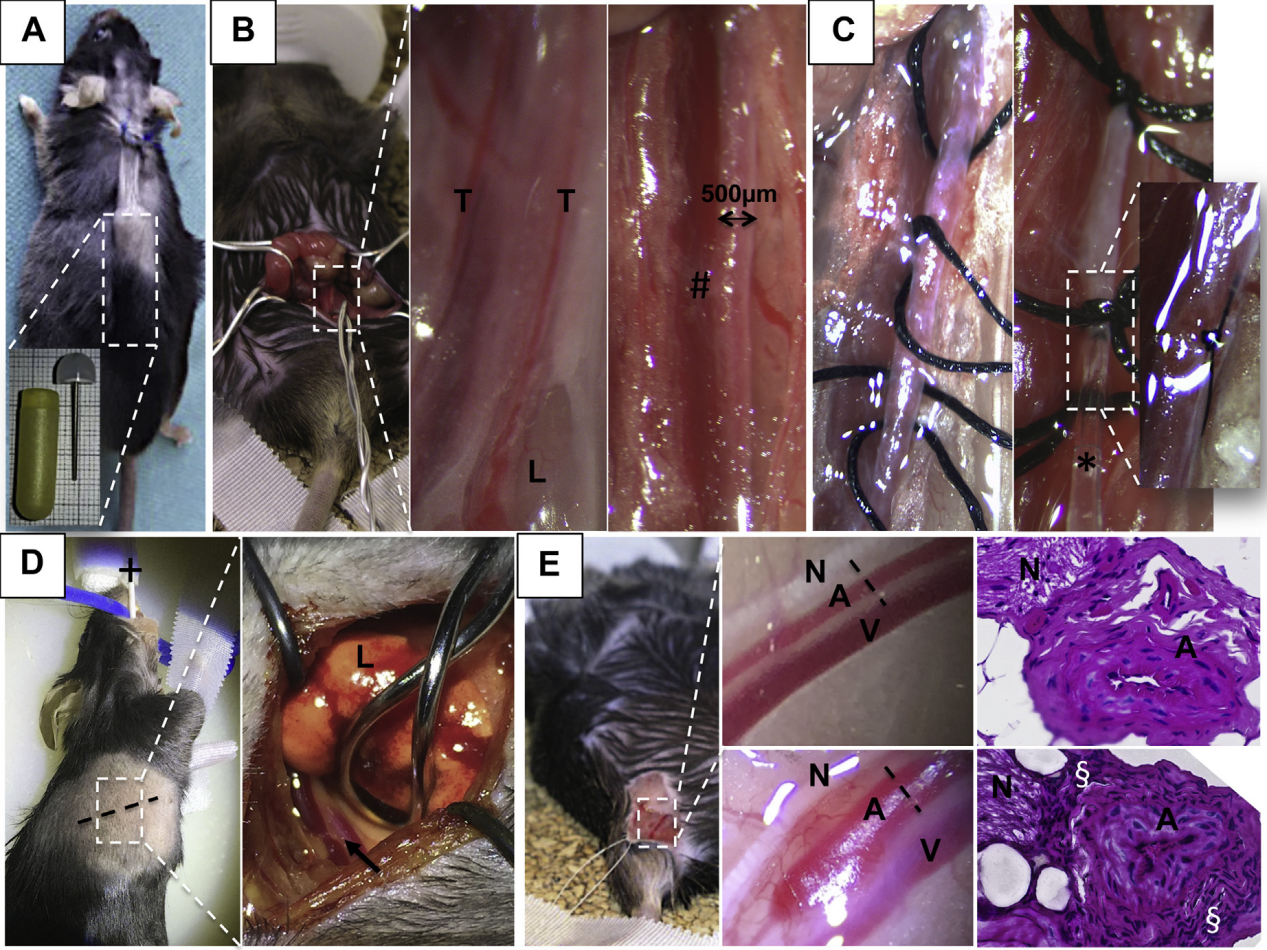
Surgical review of inducible murine aortic models. For the angiotensin II model (*AngII*), a subcutaneous tunnel is prepared through a small dorsal flank incision for the osmotic minipump (approximately 20 × 6 mm; blow-up) to gradually release the AngII over 28 days or more **(A)**. For the other models, the mouse is put in a supine position and via a transabdominal incision the retroperitoneum and the aorta is exposed (blow-up) **(B)**. The aorta is freed from its covering fascia in between the testicular arteries (*T*) and separated from the inferior vena cava (#) for topical soaking in elastase (external periadventitial elastase application [*ePPE*]) or CaCl_2_. For the PPE procedure, the aorta is prepared from the surrounding tissue circumferentially and temporary silk ligatures are placed for the insertion of the perfusion catheter (★) **(C)**. Before restoration of the blood flow, this hole is closed with a 10-0 suture (magnified subfigure) **(C)**. Exposure of the descending aorta (*arrow*) is achieved in intubated and ventilated mice (+) in a right lateral position after lateral thoracotomy (*dotted line*) and careful retraction of the left lung (*L*) (blow-up) **(D)**. Similarly, aneurysm formation of the femoral artery is achieved by exposure of the artery (*A*) and separation from the femoral nerve (*N*) and vein (*V*) (upper blow-up). Topical application of elastase leads to diameter dilation from 200 to 450 μm after 2 weeks (lower blow-up) **(E)**. Corresponding histologic sections (*dotted black line*) from these two different time points shows an increasing cellular density surrounding the artery (§).

Microsurgery in mice is challenging and the learning curve is steep. In regards to the models presented elsewhere in this article, the extent of surgical techniques ranges from (1) the fairly easy subcutaneous implantation of an osmotic minipump for the AngII model, to (2) transperitoneal exposure of the infrarenal aorta for topical application of CaCl_2_ or ePPE, to (3) the more challenging temporary ligation of the aorta and its side branches and the insertion of a catheter for intra-aortic elastase perfusion (PPE) (Fig 4, *A*-*C*). Topical application of elastase has also been used in other regions of the vasculature, including the thoracic aorta or the femoral artery, given the lower invasiveness of the procedure (Fig 4, *D* and *E*). Intrathecal injections have also been used to induce cranial aneurysms.^55^^,^^56^

(Individual photographs and histologic images are courtesy of the authors.)

Thus, the procedure time ranges from 10 minutes for AngII to approximately 55 minutes for PPE (own data). Accordingly, the technical success rate declines with increasing complexity of the model. For the inducing chemicals (ie, PPE), a variety of concentrations have been reported by different groups ranging from 0.07 to 4.5 U/mL, with some authors not including concentrations or product specifications at all.^42^^,^^57^, ^58^, ^59^, ^60^, ^61^, ^62^, ^63^, ^64^ This factor has to be taken into account when considering the outcomes of different studies (see reviews listed under Pathomechanisms). For example, in the PPE model, higher concentrations of elastase do not increase the aneurysm diameter, however, the processes occurring in the aortic wall might differ (Fig 1).^57^ Also, the incidence of aneurysm/dissection in AngII is dose dependent.^30^

Our groups currently use 2 U/mL of elastase for PPE, 30 U/mL of elastase for ePPE (based on a dilution curve experiment), 0.5 mol/L of CaCl_2_, and 1 μg/kg/minute of AngII.^32^^,^^33^^,^^44^^,^^65^ BAPN is administered at 2 g/L and available ad libitum in the drinking water.^49^

## Imaging modalities in murine AAA

**Fig 5:**
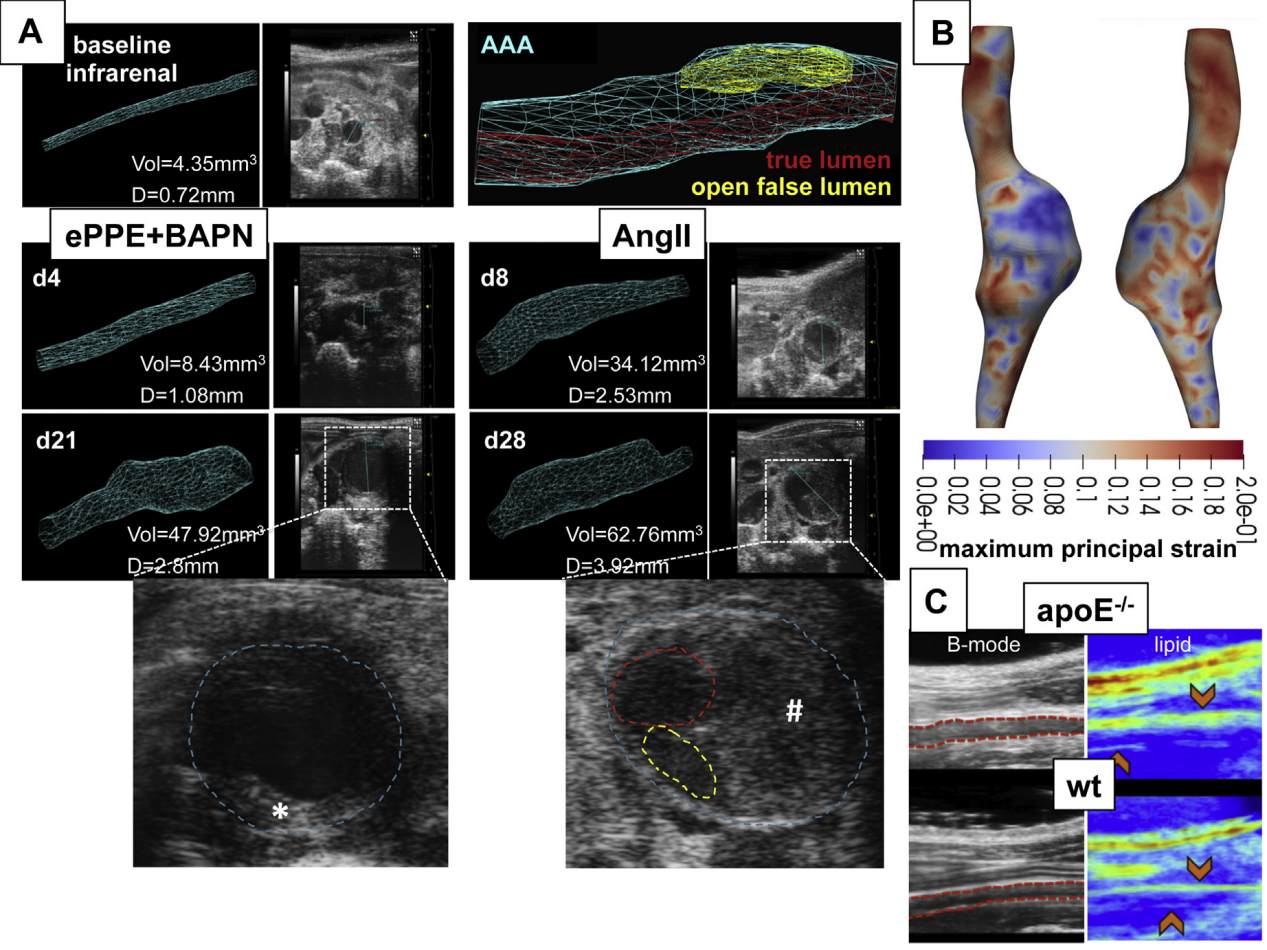
Example data from advanced imaging approaches of the murine abdominal aorta. Volumetric high frequency ultrasound of external periadventitial elastase application (*ePPE*+) β-aminopropionitrile (*BAPN*) infrarenal abdominal aortic aneurysm (*AAA*) with intraluminal thrombus (ILT) (**★**) and angiotensin II model (AngII) suprarenal dissections with intramural thrombus (**#**), true lumen (red), and open false lumen (*yellow*) (12-mm aortic length measured; outer aortic diameter. *D*, diameter; *d*, postoperative day after aneurysm induction; *Vol*, Volume. **(A)**.^36^ Three-dimensional (3D) data can be used to quantify AAA volume and allows for flexibility when measuring the maximum AAA diameter or length. The four-dimensional ultrasound data can be used to create cyclic strain maps using a direct deformation estimation approach that calculates the full 3D Green Lagrange strain tensor. In this AngII dissection, lower maximum first principal component strain values are observed within the dissection where higher amounts of collagen and intramural thrombus were present (blue) **(B)**.^68^ Vibrational photoacoustic images of the infrarenal aorta uses endogenous contrast and reveals greater perivascular lipid accumulation in apolipoprotein E-deficient (*ApoE*^*-/-*^) mice compared to wild-type (wt) **(C)**.^69^

The use of advanced, noninvasive imaging capable of producing high-resolution image data to track AAA development and expansion in mice has become fairly commonplace. For example, ultrasound imaging is a common approach that is noninvasive, reproducible, relatively fast, and widely available, making it ideal for imaging the vasculature.^66^ Clinical and preclinical ultrasound examination use almost identical approaches, with the exception that preclinical systems use much higher frequencies to produce images with a higher spatial resolution at the cost of a decreased penetration depth.^67^ Fortunately, the small body size of mice makes limited penetration depth a nonissue. Doppler ultrasound examination can be used to measure blood flow velocity (pulsed wave and color), M-mode ultrasound examination can be used to quantify circumferential strain (both linear and Green Lagrange strain), and volumetric ultrasound examination can be particularly useful when visualizing complex lesions with large tortuosity and/or false lumens (Fig 5).^36^^,^^70^^,^^71^ A relatively new imaging technique called photoacoustic imaging combines pulsed laser light with ultrasound detection to create compositional images.^72^ Photoacoustic techniques, both with and without contrast, have been shown to have usefulness in the imaging of atherosclerosis, but have not yet been applied to AAAs.^69^

(Images are courtesy of the authors and modified from the cited own publications.)

Another robust imaging strategy, small animal microcomputed tomography, can produce high-resolution three-dimensional images of the aorta and measure diameter with more precision than ultrasound examination, but requires both a vascular contrast agent, affecting renal function, and the use of ionizing radiation that might cause radiation side effects in specific cases, especially when serial imaging is done.^73^^,^^74^ Vascular computed tomography contrast agents for small animals have to be modified from clinical computed tomography contrast owing to the fast murine heart rate and blood clearance times in mice. After humane killing, microcomputed tomography has been shown to be useful when detecting microbreaks within the wall associated with branch ostia.^75^^,^^76^

Further, high-field magnetic resonance imaging using small bore systems have also shown usefulness for imaging murine AAAs.^77^^,^^78^ Common techniques include time of flight, which takes advantage of blood flow into aneurysms, gadolinium-enhanced magnetic resonance imaging, black-blood spin-echo sequences for imaging of the aortic wall, and a variety of novel imaging contrast agents for specific markers.^79^ When using AngII-filled osmotic pumps, users typically need to replace the stainless steel flow modulator with one made of polyethylene plastic that will not create an image artifact (www.alzet.com/guide-to-use/mri/). Finally, optical imaging has become a common alternative small animal imaging approach vs positron emission tomography or single photon emission computed tomography, which are more common in clinical imaging, although all three have been used to study murine AAAs.^80^, ^81^, ^82^

Taken together, the growing body of noninvasive small animal imaging literature provides evidence that these strategies can help to decrease the total number of animals used, provide additional information regarding AAA progression, and help with translational approaches to the clinic where similar imaging techniques are used.

## Modifications of mouse models to meet human mimicry

Although AAA is a multifactorial disease, it has been associated with clearly defined risk factors for its occurrence and its progression.^26^ These factors include smoking, family history, hyperlipidemia, hypertension (metabolic syndrome), male sex, and Caucasian origin.^4^ In contrast, diabetes, if treated with metformin, seems to be protective for AAA development.^83^^,^^84^ Thus, the question remains how well these established risk factors are reflected in murine models. Table III summarizes the currently available evidence.

**Table III:** Summary of how animal models reflect risk factors and observations from human disease

Risk factor	Significance in human AAA	Model	Reflection in murine models
Age	Age-dependent increasing incidence of AAA^4^	PPE AngII	Mainly 10 week-old-mice treated^11^^,^^23^^,^^32^^,^^85^; bigger AAA in PPE in older mice (unpublished); higher incidence of AngII dissection in older animals^30^
Family history	Higher prevalence if one sibling is affected, higher in twins^86^	–	Not applicable
Ethnicity	Highest prevalence in Caucasians^87^	ePPE AngII	Different susceptibilities to the induction stimuli in mice from different strains (ie, size, VSMC biology)^88^^,^^89^
Smoking	Major risk factor for AAA and associated with faster aneurysm growth^4^^,^^87^	AngIIPPE	Nicotine instigates AAA formation, increases the incidence (AngII) and promotes AAA growth, rupture. and arterial stiffness^45^^,^^57^^,^^85^^,^^90^, ^91^, ^92^, ^93^, ^94^, ^95^
Male sex	Higher AAA incidence in men, worse outcomes after aortic repair in women^26^^,^^96^	AngII PPE	Aneurysm formation 5-10 times higher in male mice^97^ Female sex hormones prevent AAA formation^98^
Hypertensive disease	Major risk factor for AAA	AngII	Aneurysm formation/dissection is not associated with hypertension^99^
Metabolic syndrome	No direct association	AngII	AAA incidence 3-4 times higher in hypercholesterolemic mice^53^^,^^100^
Connective tissue disease	Variety of aortic and other aneurysms in patients with a variety of CTD^101^	–	Individual mouse models with specific mutations, but no combination with AAA models^102^
Observation			
Disturbed/altered iliac outflow	Progressive AAA kinking and higher AAA incidence^103^	PPE	Promotes AAA kinking and catalyzes potential adventitial angiogenesis^32^^,^^104^
Metformin	possibly protective of AAA^105^	AngII	slows AAA/dissection growth^106^^,^^107^
Statins	Statins reduce AAA growth and AAA-related mortality^4^^,^^26^	AngII PPE	Statins suppress AAA formation in normal and hypercholesterolemic mice^108^^,^^109^
Platelet inhibitors	Recommended in patients with AAA to lower cardiovascular comorbidities^4^	AngII	Decreased the increase of aortic diameter, leukocyte infiltration, MMP9 expression and elastic fiber degradation^110^^,^^111^
ILT	Viscoelastic, biologically active thrombus observed in >90% of AAAs^26^	ePPE	Observed regularly, currently no data on biological activity^12^
Atherosclerotic aorta	High coincidence of atherosclerosis and AAA in human, yet considered distinct diseases^52^	AngII CaCl_2_ PPE ePPE	See section above on the significance of the atherosclerotic murine aorta
AAA localization	>80% infrarenal^26^	AngII CaCl_2_ PPE ePPE	AngII aortic dissections are seen mostly at the ascending and thoracoabdominal portion of the aorta proximal to the renal arteries^29^ for the other models, the localization is subject to surgical exposure (Fig 4)
Rupture	Most frequently contained into retroperitoneal space^4^	AngII ePPE	AngII: open rupture or intramural bleeding^29^ ePPE: open rupture or contained to retroperitoneal space^12^

*AAA*, Abdominal aortic aneurysm; *AngII*, angiotensin II; *CTD*, connective tissue disease; *ePPE*, external periadventitial elastase application; *ILT*, intraluminal thrombus; *MMP*, matrix metalloproteinase; *PPE*, periadventitial elastase application; *VSMC*, vascular smooth muscle cell.

Additionally, genome-wide association studies have identified a small number of risk alleles, possibly influencing AAA that are still widely underinvestigated, even in human disease.^26^ In mice, genome-wide analysis comparing AAA models with their untreated littermates are largely missing and would probably only make sense in mice with spontaneous AAA (Tables I and III).

## A critical appraisal for study design involving AAA animal models

According to the American Anti-Vivisection Society, as many as 93% of animals used in experiments in the United States are mice and rats, roughly 25 to 100 million each year. In Germany, approximately 2.1 million mice are used each year, accounting for 75% of all laboratory animals.^112^ Although AAA research might only be a small percentage of these totals, animal welfare, minimizing the use of research animals, and good scientific practices should always be guiding principles.

Further important considerations for the future use of AAA animal models based on our personal experience are highlighted as bullet points and should spur additional discussion.

• Reporting standards, currently exerted for many vascular pathologies by the European Society of Vascular and Endovascular Surgery and the Society of Vascular Surgery should also apply to murine AAA research.^113^^,^^114^ For example, the greatest heterogeneity is found in treatment studies from the AngII model, where “dissection, aneurysm and rupture” are often combined. Unfortunately, the classification system presented for reporting outcomes remains largely unrecognized.^29^ Similarly, ultrasound examination of the murine aorta should be standardized applying, for example, the “leading-edge method” in analogy to human ultrasound modalities.^4^^,^^115^^,^^116^ Finally, thrombus should be clearly labeled as intraluminal (as in human AAA) or intramural (as in false lumen thrombosis in dissection) (Figs 2 and 3)
• Although the AngII model is considered the best investigated, it is also the most complex model in terms of outcome measurements and reports (Fig 1).^29^^,^^30^ Further meta-analyses exist for CaCl_2_, but are missing for PPE and ePPE.^31^ Considering the huge microsurgical effort for the PPE model, a systematic review of the data might be helpful to improve its further application. Additionally, methodological studies of the timing associated with aortic events reflecting the features of human disease are still needed (Table II), especially in the newer models. Currently, most mechanistic insights are drawn from interventional studies with some drug of interest.
• The growth of the aneurysmal lesion is relatively rapid in all of the models and thus differs significantly from human disease, which is characterized by chronic development (Fig 1). In fact, a vascular “healing response,” implying clearance of inflammatory processes, is observed in most models. Some lesions may even regress in diameter 4 to 8 weeks after AAA induction.^31^^,^^32^ Thus, knowledge about the specific events during AAA formation is crucial for the correct timing of interventional studies with novel therapeutics of interest (Fig 1). The status quo of treatment initiation alongside AAA induction is probably biased, since at the early stages, none of the characteristic features of human disease (Table II) are mimicked and only acute inflammation is treated.
• The PPE model is in many reviews and, in our opinion, accepted to be the best mimicry of human AAA disease.^23^^,^^26^^,^^117^ Owing to its surgical nature, it can be modified by eg iliac ligation to trigger aneurysm bulging or even to juxtarenal or suprarenal aneurysm formation by careful dissection of the visceral segment.^32^^,^^104^^,^^118^ Additionally, the model has been successfully transferred into a preclinical pig model, adding a second species with human-relevant physiologic features by others as well as our group.^119^^,^^120^ However, the surgical learning curve is slow and no ILT is observed in this model.

## The translational research needs of vascular surgery: unmet research questions

In vascular surgery, a couple of unmet questions remain open to be addressed by translational research. Yet the question remains if and how our current models can help to translate basic science findings into clinical therapeutics.

### The origin and cause of AAA development

Why do the majority of aneurysms occur at the abdominal part of the aorta? Studies have suggested disturbed flow and higher pressure wave reflections at the aortic bifurcation, probably in combination with previous atherosclerosis leading to endothelial “microdamage,” propagating medial disruption.^121^^,^^122^ Second, the composition of the aortic wall, that is, the elastin content or collagen types, differs from the ascending aorta to the bifurcation.^123^ Such conditions could also cause initial thrombus formation according to Virchow's triad.^124^ Additionally, the infrarenal aorta is primarily prone to stiffening effects, which in turn establishes a stiffness gradient between adjacent aortic segments.^125^ Alternatively, a unique responsiveness to various stimuli within the infrarenal aorta, such as transforming growth factor-β signaling both early in embryogenesis and also later during adult life, has been previously reported.^37^^,^^126^

Because all of the inducible models have a defined external stimulus, resulting in regressive rather than exponential AAA growth, mouse models might not be able to answer why aortic aneurysms develop predominantly in the infrarenal region (Table I). However, studying the effects of these various stimuli to distinct aortic segments (ie, ascending, thoracic, suprarenal, infrarenal) could further elucidate the role of different aortic segments based on embryological originality.

## Sex aspects in AAA occurrence and treatment

Epidemiologic studies reveal that AAA occurs less frequent and later in life in women (approximately 9:1) and that women have higher rupture risk and a worse outcome after surgery (Table III).^127^^,^^128^ The outcome is mostly attributed to poorer preoperative health status, less frequent suitability for endovascular aortic repair and the fact that rupture risk based on aneurysm diameter might not be comparable to men owing to different body metrics (height, weight, body mass index, and body surface area).^4^^,^^127^^,^^129^ Villard et al^130^ have performed a systematic review on AAA and sex aspects and concluded that gender might influence AAA development, but current data is insufficient to support a biological role of sex hormones.

Although some studies for sex differences and the influences of sex hormones on AAA development have been performed in mouse models (Table III), basically no interventional studies have investigated male and female mice separately on the outcome of treatment. Thus, for future tests of medical treatments, male and female mice should be part of the study.

### Modified surgical therapies

Although endovascular aortic repair is well-established as the treatment of choice for most infrarenal AAAs, new and improved devices will always require testing.^4^ In the future, biologically active endovascular aortic therapies (ie, drug-eluting techniques) might be of relevance. Owing to size, endovascular treatments in mice have always been very limited. However, Baker et al^131^ and Chamberlain et al^132^ have published on direct aortic and even transfemoral implantation of bare metal stents (yet no stent grafts) in mice. Our group has previously established AAA treatment in PPE by reperfusion of the AAA on day 7 after induction via a second laparotomy (unpublished data).

Regardless of size, mice can be used for endovascular treatment options in interventional studies, even with device implantation on an experimental basis.

### Role of the ILT on AAA development

ILT occurs in about 80% of human AAAs and often has a complex multilayered structure.^133^ On the one hand, the ILT is proposed to decrease peak wall stress and thereby protect the aneurysm from rupture.^134^^,^^135^ In contrast, the ILT represents an active site of leukocyte entrapment and activation, which enhances the local release of wall-destructive molecules (such as reactive oxygen species or proteases) and thereby promotes vessel degradation.^136^^,^^137^ A multitude of canaliculi allows passive and active transport between the ILT and the aortic wall.^138^ The amount of proteases and plasma proteins found within the aortic wall correlates strongly with AAA growth; however, a decreasing gradient of active enzymes from inner to outer layers must be considered.^139^^,^^140^ Small clinical trials showed that platelet activation contributes to AAA development and progression as antiplatelet medication (aspirin) was beneficial in terms aneurysm size and less frequent surgical repair.^141^

Generally underinvestigated in human disease, most of the mouse models do not recapitulate this ILT formation. Promising results come from ePPE, probably in combination with BAPN (Figs 2 and 5). Further biomechanical and inflammation assessment of this thrombus should be undertaken to elucidate its specific effect on AAA growth. Additionally, some human aneurysms show intramural hemorrhage as a pathological feature, which is mimicked by the intramural thrombus in the AngII model to some extent (Fig 2).

### AAA risk assessment based on monitoring aneurysm growth, stress, and flow analysis

The current European Society of Vascular and Endovascular Surgery guideline weakly suggests risk assessment based on possibly available stress and flow analysis tools to support the indication for surgical therapy.^4^ Many different techniques, such as speckle tracking, finite element analysis, and flow simulation have been investigated with reliable results, suggesting that integrating biomechanics-based diagnostic indices may significantly help in decision-making; however, it is currently a time-consuming process and needs refinement before broad clinical application.^26^^,^^142^

Mouse models, especially the potential of reliable ILT formation and eventual rupture in ePPE (+BAPN) in combination with advanced imaging techniques will be helpful in the future to monitor aneurysm growth, analyze wall stress and correlate this with biological changes in the aortic wall (Fig 5).

### Novel nonsurgical treatments

Currently, many promising alternative treatment strategies are being investigated in the context of AAA, including noncoding RNA therapies, drug repurposing strategies, and more sophisticated strategies such as exosome-based drug delivery.^143^, ^144^, ^145^ Most of these are considered experimental, yet will require in vivo testing at some point.

Mice remain the gold standard of translational testing for experimental therapies. Thus, considering all these benefits, AAA mouse models will likely have a significant role assessing multiple methods of drug administration (ie, by intravenous miniport, intraperitoneal, systemic, and local endovascular delivery).

## Conclusions

• AAA mouse models are valuable tools for translational research, each mimicking specific features of human disease.
• Although AngII is currently the best-investigated model, its most prominent feature is aortic dissection with intramural hemorrhage developing between the media and the adventitia. Reporting standards should be consistent and maintained.
• PPE might produce the best mimicry of human disease, however, it is surgically challenging and does not feature ILT or rupture.
• Both of these disadvantages are overcome in ePPE, especially in combination with BAPN, but good mechanistic insights are yet to be established.
• The specific timeline of AAA development dependent changes in the aortic wall is crucial for reproducible translational results.
• Modern high-resolution imaging techniques, the possibility of endovascular treatment, and novel nonsurgical therapy options provide an armamentarium for excellent future AAA studies involving mouse models.

## Author contributions

Conception and design: AB, SB, NI, MW, HE, CB, MW, CG, LM

Analysis and interpretation: Not applicable

Data collection: Not applicable

Writing the article: AB, SB, CG, LM

Critical revision of the article: AB, SB, NI, MW, HE, CB, MW, CG, LM

Final approval of the article: AB, SB, NI, MW, HE, CB, MW, CG, LM

Statistical analysis: Not applicable

Obtained funding: Not applicable

Overall responsibility: AB

AB and SB contributed equally to this article and share co-first authorship.
